# Psychosocial vulnerability underlying four common unhealthy behaviours in 15–16-year-old Swedish adolescents: a cross-sectional study

**DOI:** 10.1186/s40359-017-0209-9

**Published:** 2017-12-15

**Authors:** Ulrica Paulsson-Do, Birgitta Edlund, Christina Stenhammar, Ragnar Westerling

**Affiliations:** 1 Department of Public Health and Caring Sciences, Section for Sociomedical Epidemiological Research, Uppsala University, Uppsala, Sweden; 2 Department of Public Health and Caring Sciences, Caring Sciences, Uppsala University, Uppsala, Sweden; 3 Department of Public Health and Caring Sciences, BMC, Box 564, 751 22 Uppsala, Sweden

**Keywords:** Adolescents, Unhealthy behaviours, Vulnerability, Social relationships, Self-esteem, Well-being

## Abstract

**Background:**

Factors that influence unhealthy behaviours in adolescents may have different impacts in different sociocultural settings. There is lack of research on the association between psychosocial vulnerability and unhealthy behaviours in adolescents, particularly outside the United States. The aim was to investigate both direct and indirect relationships between psychosocial conditions (subjective well-being, social relationships and self-esteem) and four health-related behaviours (smoking, alcohol consumption, meal frequency and physical activity) in Swedish adolescents aged 15–16 years. Socio-demographic variables (socio-economic status, gender and age) were also investigated.

**Methods:**

To study these associations, a hypothesised model was tested using structural equation modelling. In the hypothesised model, interrelated psychosocial conditions (low well-being, poor social relationships and low self-esteem) and socio-demographic factors (low self-perceived socio-economic status, being female and higher age) together represented a vulnerability underlying smoking, alcohol consumption, irregular meal frequency and low level of physical activity. In this cross-sectional study, self-report questionnaires were used to collect data from 492 adolescents.

**Results:**

Hypothesised pathways between psychosocial conditions, socio-demographic factors and the four unhealthy behaviours were confirmed. Low well-being was strongly associated with unhealthy behaviours, and poor social relationships showed a strong indirect association with the unhealthy behaviours. Low self-esteem, low self-perceived socio-economic status and female gender were also vulnerability factors for the unhealthy behaviours.

**Conclusions:**

Vulnerability for four common unhealthy behaviours was found in Swedish adolescents. This study presents the interrelationships of psychosocial and socio-demographic factors and how they were related with unhealthy behaviours. The results bring new insight into how psychosocial factors are related to unhealthy behaviours in adolescents living in northern Europe.

**Electronic supplementary material:**

The online version of this article (10.1186/s40359-017-0209-9) contains supplementary material, which is available to authorized users.

## Background

Unhealthy behaviours have outpaced infectious disease as the primary cause of death in industrialised countries [1]. Smoking, alcohol abuse, unhealthy eating habits and low level of physical activity are great risk factors for a large number of serious conditions and diseases [2]. Meal habits, for example, have been found to be associated with both mental and physical health [3] and are known to be associated with overweight in adolescents [4–6]. As adolescence is a critical period of life when health-related behaviours are set for adulthood [2, 7], it is crucial that adolescents have healthy behaviours.

Adolescence is a period of maturation during which adolescents have to deal with changing social relationships [2] with both family and peers [8]. These changing relationships may influence adolescents’ health-related behaviours. Studies have found social relationships with family [9, 10] and peers [10, 11], as well as self-esteem [10, 12, 13], to be important to a person’s well-being. The research literature has also identified low well-being [14, 15], poor social relationships [16, 17] and low self-esteem [9, 16, 18] to be psychosocial causes of unhealthy behaviours among adolescents. While most of these studies investigated the relationships of individual factors to unhealthy behaviours, increasing number of studies have acknowledged the multifaceted nature of underlying factors of unhealthy behaviours [19].

Jessor and colleagues [20–23] performed studies on adolescents in the United States, examining so-called ‘problem behaviours’ (i.e. typical health-compromising behaviours), including alcohol consumption, drug use, precocious sexual intercourse and delinquency. They investigated a wide range of possible underlying factors to these behaviours, including low self-esteem, social isolation and unhappiness, among others [23], and found a grouping of factors that increase involvement in ‘problem behaviours’ [20, 23]. A few similar studies followed that investigated multiple underlying psychosocial factors of health-related behaviours in adolescents [9, 17, 19, 24]. Wiefferink and colleagues performed a systematic review that aimed to identify the extent to which health-related behaviours cluster and whether their determinants were associated. They found that unhealthy behaviours cluster in adolescents and that they share multiple underlying factors, including low self-esteem and poor psychosocial relationships [16]. The grouping of underlying factors may be explained as being a vulnerability that can increase involvement in unhealthy behaviours [19] or, as defined by Blum and Blum [25], vulnerability may be explained as a state, which is generated from the presence of factors that increase the risk for unhealthy behaviours. Research investigating the notion of a vulnerability that contributes to several unhealthy behaviours in adolescents is sparse, however, and most of the existing studies and theoretical models are performed in the United States, whereas few have been conducted in Europe [16]. The American ‘Individual Health Behaviours Model’, as presented by Shi and Stevens [19], builds upon ideas which were first recognised in a report by the Minister of Health in Canada in 1974 [26]. The theoretical model proposes that people with a vulnerability engage in more health-compromising behaviours, such as smoking and alcohol consumption than in health-enhancing behaviours, such as healthy eating and regular physical activity. The ‘Individual Health Behaviours Model’ argues that poor social relationships as well as low self-esteem cause low well-being, which leads to unhealthy behaviours [19]. Although several publications report associations between health-related behaviours and well-being, social relationships and self-esteem [8, 9, 16, 27], the ‘Individual Health Behaviours Model’ has not been scientifically tested.

The degree to which psychosocial factors are important to well-being and health-related behaviours may be related to the welfare regime used in a country. In countries with the conservative system (such as Germany) [28] or the liberal system (such as the United States [28]and the United Kingdom [29]), the state does not provide full social rights as countries using the social democratic model (also called the “Scandinavian model”) (such as Sweden) [28]. There are health inequalities between countries with different welfare regimes in Europe [30, 31]. Well-being [32] and health-related behaviours [27, 33] are generally good in Swedish adolescents, for example, as opposed to other countries, such as the United Kingdom [33]. Rostila [31] found social capital to be an explanation for health inequalities between countries with different welfare regimes.

**Fig. 1:**
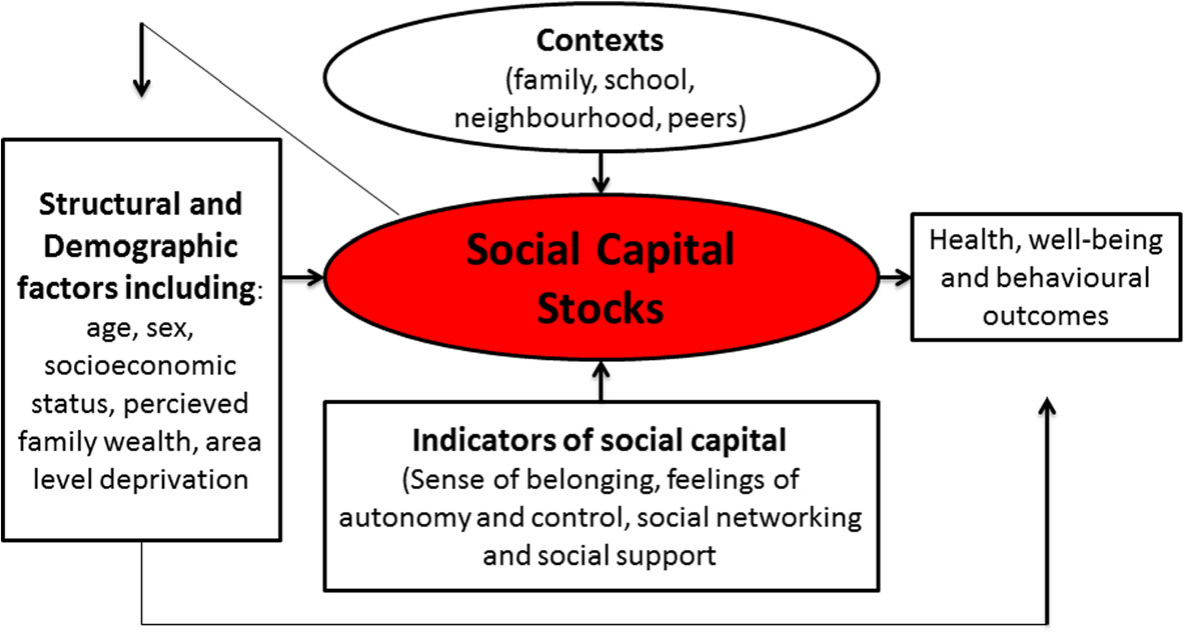
Morgan’s social capital framework

Morgan [9] reported on a framework, which was scientifically tested and got empirical support. It was used to analyse underlying psychosocial and socio-demographic factors (or as he termed, “social capital stocks”) of adolescents’ health-related behaviours, well-being and health (Fig. 1). The framework was built upon collected scientific evidence [34–36] and aimed to demonstrate that social capital could be protective of the well-being and health-related behaviours of adolescents. Morgan used the framework to study Spanish and English adolescents. The psychosocial and socio-demographic factors were found to be associated with adolescents’ well-being, health-related behaviours and health in both countries. However, his findings suggested that factors for unhealthy behaviours may have different impacts in different socio-cultural settings. In particular, the degree to which psychosocial factors were important to well-being and health-related behaviours differed between the two countries. For example, social support appeared to be a more prominent factor for well-being in Spain compared with England. Morgan argued that this difference could be explained by cultural “values in Spain that encourage interdependence and leans more towards the collective rather than the individual” [37]. Morgan [9] also found that the configuration of how underlying factors were associated with well-being and health-related behaviours differed between Spanish and English adolescents. Studies of the interrelation of underlying psychosocial conditions to unhealthy behaviours are sparse. Such studies are needed to determine how different psychosocial components are related to health-related behaviours of adolescents. Socio-demographic groups that are more prone to poor psychosocial conditions underlying unhealthy behaviours are important to identify, for example.

Paulsson-Do and colleagues investigated a hypothesised model of a set of unhealthy behaviours using structural equation modelling in northern Europe [38]. The hypothesised model, that was tested, was based on earlier research findings [38] and was supported empirically. The study included Swedish adolescents and found that smoking, alcohol consumption, irregular meal frequency and low level of physical activity shared an underlying vulnerability. The study focused on socio-demographic factors and found, similar to other studies, that socio-economic status [39–42], gender [15, 39, 43] and age [41–44] were vulnerability factors associated with unhealthy behaviours in adolescents. The study did not, however, investigate poor psychosocial conditions and their possible interrelations with socio-demographic groups and an underlying vulnerability to unhealthy behaviours.

Although, few studies have investigated the relationships between psychosocial conditions and socio-demographic groups, studies have demonstrated that girls have lower self-esteem than boys [45–47] and that boys sometimes experience better social relationships than girls [48, 49]. Studies have found low socio-economic status to be related with lower self-esteem [14, 50, 51] and low well-being [52, 53]. Well-being has also been found to be connected with self-esteem [10, 12, 13], social relationships [10, 12, 13] and health-related behaviours [8, 9, 54]. These associations have not been investigated in relation to an underlying vulnerability of unhealthy behaviours in northern European adolescents.

**Fig. 2:**
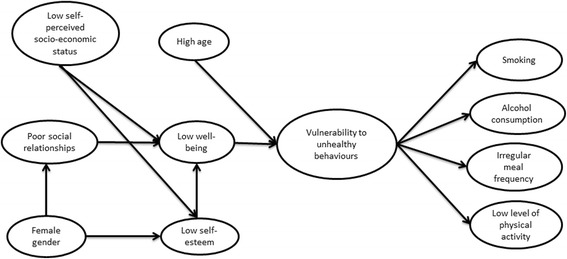
Hypothesised path model. Hypothesised path model of interrelated factors composing a vulnerability to the unhealthy behaviours smoking, alcohol consumption, low level of physical activity and irregular meal frequency in Swedish adolescents aged 15 to 16 years. First-order latent variables are presented in squares. The second-order latent variable is presented in the oval circle

The present study aimed to contribute to the development of a framework of underlying direct and indirect psychosocial (subjective well-being, social relationships and self-esteem) associations with health-related behaviours (smoking, alcohol consumption, meal frequency and physical activity) in adolescents (aged 15–16 years) in Sweden. This study proposed a new integrated theoretical model (Fig. 2), which was further developed from the ‘Individual Health Behaviours Model’, Morgan’s social capital framework (Fig.1) and the findings from the structural equation model studied by Paulsson Do and colleagues. The proposed theoretical model was also developed from earlier research findings regarding associations between psychosocial conditions and socio-demographic groups. The model tested whether interrelated poor psychosocial conditions and socio-demographic factors (low self-perceived socio-economic status, being female and older age) together represent vulnerability to smoking, alcohol consumption, irregular meal frequency and low level of physical activity.

## Methods

### Sample

All municipal upper schools (161 schools) in 21 Swedish municipalities (out of 290 municipalities in Sweden) were asked to participate in the study. These municipalities were strategically selected to ensure a geographical and socio-demographical spread among the participants in the study. The selected municipalities included both urban (city with a population of 50,000 or more or a municipality near a city of this size where a high percentage commute to the city) and non-urban-populated municipalities (less than 50,000 inhabitants and not a municipality near a city of at least 50,000 inhabitants where a high percentage commute to the city) as defined by the Swedish Association of Local Authorities and Regions [55]. Nineteen schools in 14 municipalities agreed to participate. Ten schools (in nine municipalities) were chosen, representing equal distribution of schools and pupils in urban and non-urban-populated municipalities, which were geographically spread out (805 registered pupils, 412 in non-urban-populated and 393 in urban-populated municipalities). The final sample in the study consisted of 492 pupils (61%) (in school grades 8 and 9 (ages 15–16 years) from compulsory school). Non-responses were owed to one school failing to return their questionnaires (123 questionnaires), teachers failing to distribute the questionnaires (151 questionnaires) and pupils who were absent on the particular day that the questionnaire was distributed (39 pupils). Out of the 492 pupils, 240 adolescents lived in urban-populated municipalities (49%) and 252 adolescents in non-urban-populated municipalities (51%). This sample is representative of the Swedish population with regard to geographical spread, education level of parents and number of pupils with passing certificates upon graduation from grade 9 [55–57].

### Procedure

An 82-item questionnaire was handed out to pupil participants who completed it using paper and pencil (in approximately 30 min) in the classroom during class hours. The questions were divided into subsections with different subheadings. Twenty-six of the questions were used in this cross-sectional study (see Additional file 1). Twenty-five of these questions had been used in previous studies and one was developed for this study (‘self-perceived economic situation’). To ensure the validity of the questions in the questionnaire, a pilot study was conducted in a school class (grade 9). This school did not provide data for the current study. A test–retest was performed on the same school class. A few questions and answering alternatives were then reformulated. A second pilot study was performed in four school classes to test the validity of the edited questions. The test–retest was performed to ensure reliability of the questions in the questionnaire that were developed by the research team. The test–retest analysis was performed in SPSS Statistics (version 17.0) using cross-tabulation and Spearman correlation and the reliability was found to be adequate (Correlation coefficients ranging from 0.6 to 0.9).

### Ethics, consent and permissions

The first page of the questionnaire provided information about the study’s aim, confidentiality, informed consent (which was given by answering the questionnaire), voluntary participation and the option to withdraw from the study at any time. Questionnaires were returned in June 2009.

The Swedish law of ethical regulations and guidelines for humanistic and social science research [58] were followed in this study. The study was performed according to the Declaration of Helsinki and the ethical standards of the ethics committee at the Faculty of Medicine at Uppsala University, Sweden. Following ethical standards and this law (Law, 2003:460), the ethics confirmed that the study was exempt from requiring ethical approval.

### Study variables

Study variables included health-related behavioural variables, psychosocial condition variables and socio-demographic variables (Additional file 1).

#### Variables

##### Health-related behavioural variables

Meal frequency was selected to measure eating habits. Three items assessed meal frequency (‘How often do you eat the following meal during a regular week? Breakfast? Cooked lunch? Cooked food in the evening?’ (Cronbach’s Alpha 0.671) (Additional file 1)). One question for each health-related behaviour was used for the other health-related behaviours: ‘How often do you exercise in your spare time for more than 30 minutes so that you get out of breath or sweat?’ ‘Do you smoke?’ and ‘Do you drink alcohol so that you become drunk?’

##### Psychosocial condition variables

Subjective well-being was measured by two commonly used variables [59, 60] (‘How do you feel?’ and ‘How satisfied are you with your life?’) (Cronbach’s Alpha 0.800). Social relationships were measured using the interpersonal distrust subscale (7 questions) from the Eating Disorder Inventory-Children (EDI-C) [61], Swedish version [62–64]. The variable ‘social relationships’ (referring to the quality of relationships, for example, trust and social support) consisted of the total score for the subscale. Items measuring social relationships included, for example, ‘I trust others’ and ‘I have close friends’. Self-esteem was measured with the ineffectiveness subscale (10 questions) from the EDI-C [61], Swedish version [62–64]. The variable ‘self-esteem’ consisted of the total score on the subscale and included questions that measured factors such as feelings of autonomy and control (for example, ‘I feel that I can attain the things I try to’ and ‘I feel that I can control things in my life’). As recommended for these validated subscales [61], mean values were used to replace isolated missing responses on the interpersonal distrust subscale and ineffectiveness subscale. These subscales have good internal consistency [61] (Cronbach’s Alpha was 0.760 for the interpersonal distrust subscale and 0.881 for the ineffectiveness subscale).

##### Socio-demographic variables

Demographic variables included age and gender (Additional file 1). Adolescents’ self-perceived economic situation was also assessed (using the question ‘How often do you feel that you have less money than your peers?’); this is a good measure of socio-economic status in relation to health-related behaviours [65].

#### First-order latent variables

Health-related behaviours, psychosocial condition variables and socio-demographic variables were all analysed as first-order latent variables (constructs consisting of one or more items from the questionnaire). We first determined whether items represented latent phenomena [66]; for example, whether the items ‘How do you feel?’ and ‘How satisfied are you with your life?’ represented subjective well-being. Variables for social relationships and self-esteem were constituted by the total scores on their respective subscales ‘interpersonal distrust’ and‘ineffectiveness’ (indicating quality of social relationships and level of self-esteem) in the EDI-C [61]. These variables were then included in the analysis as first-order latent variables. To use only one indicator for a latent variable is common practise in SEM analysis when this indicator alone measures what the authors intend to measure [66].

#### Second-order latent variable

It was hypothesised that vulnerability represented a latent phenomenon underlying the tendencies to smoke, consume alcohol, eat irregularly and refrain from physical activity (Fig. 1). A second-order latent variable [67–69], which is a construct that represents several first-order latent variables [68, 69] was used to test whether there was a shared underlying psychosocial vulnerability for this set of unhealthy behaviours (i.e. smoking, alcohol consumption, irregular meal frequency and low level of physical activity level).

### Statistical analyses

The statistical programme LISREL (version 8.80) was used for measurement modelling analysis, correlation analysis and structural equation modelling (SEM) [67]. Maximum likelihood was used to deal with missing values. There were no differences in demographics, in terms of participants who were and were not missing data.

To confirm or reject whether certain indicator variables represented latent variables [66], and to examine the validity of these variables, measurement modelling was performed by confirmatory factor analysis measuring the degree to which each item significantly loaded (as indicated by path coefficients) onto its designated first-order latent variable [67]. Items measuring regular meal frequency of ‘breakfast’, ‘cooked lunch’ and ‘cooked food in the evening’ were tested to determine whether they loaded onto a first-order latent variable for ‘meal frequency’. Similarly, the items ‘How do you feel?’ and ‘How satisfied are you with your life?’ were tested to see if they were reliable measures of subjective well-being.

To confirm whether the first-order latent variables significantly correlated with a second-order latent variable (as indicated by correlation coefficients), a polychoric correlation analysis was conducted with the first-order latent variables and a second-order latent variable (see Additional file 2) in LISREL.

The definition of the unit of measurement, which has to be obtained when second-order latent variables are measured in SEM, was specified by fixing the unstandardised direct effect of the second-order latent variable and the first-order latent variable ‘smoking’ to 1.00 [67, 68]. ‘Smoking’ was chosen because it had the strongest loading with the second-order latent variable in the correlation analysis (Additional file 2) [69]. This scaling controls the remaining path coefficients between the second-order latent variable and its first-order (indicator) latent variables.

The hypothesised model (Fig. 2) was tested by SEM analysis. The total scores of the social relationships scale and the self-esteem scale measured good social relationships and high self-esteem. For this reason, as well as to make the presentation of the results more legible, the direction of variables in the SEM model were analysed as presented in Fig. 2 (i.e., high well-being, good social relationships, high self-esteem, high socio-economic status, regular meal frequency and high level of physical activity were tested in the analysis with the expectation of negative associations with the vulnerability variable).

SEM analysis was chosen because it offers certain advantages. For example, when compared with regression analysis, SEM allows the researcher to hypothesise and test the strength of relationships between first- and second-order latent variables; i.e. associations with underlying factors may be estimated [68, 69]. SEM also allows complex model analyses, including mediating associations [68, 69]. Previous studies that are similar to the present one have used second-order latent variable modelling to measure the underlying structure of unhealthy behaviours [21, 70–72]. SEM analysis determined whether the variables outlined in the hypothesised model (Fig. 1) (which was based on theoretical and empirical evidence) were associated with each other. The strength of the hypothesised associations in the model was indicated by path coefficients [67]. Total associations (direct and indirect associations) between all variables in the SEM model were tested. The total path coefficients determined the associations between the individual health-related behaviours and each psychosocial condition variable, and indicated whether the psychosocial conditions and the socio-demographic groups reflected an underlying vulnerability to the set of unhealthy behaviours included in the study. As this is a cross-sectional study, however, the path associations are not causal.

Path coefficients of associations between variables in the measurement model analysis (see Results section) and the SEM analysis (see Additional file 3) were confirmed when they were statistically significant at 95% CI. The polychoric correlation associations were confirmed when they had significant *p*-values (Additional file 2). The measurement model, correlation analysis and SEM analysis were all evaluated by fit measures. These measures indicated how closely these analyses fitted the data. Good fit is indicated by a low root-mean-square error of approximation (RMSEA, acceptable fit below 0.08), non-significant χ^2^, high Goodness of Fit Index (GFI > 0.90), high Goodness of Fit Index Adjusted for df (AGFI >0.90) and low standardised root-mean-square residual (SRMR, acceptable fit below 0.08) [73].

## Results

### Descriptive analysis

Descriptive statistics for the variables included in the model are reported in Additional file 1.

### Assessment of the measurement modelling analysis, correlation analysis and SEM analysis

The measurement model indicated a good fit of the data (*χ* ^2^ 2.28 with df = 3, RMSEA = 0.00, GFI = 1.00, AGFI = 0.99 and SRMR = 0.01). The fit statistics that assessed the plausibility of the correlations of the first-order latent variables with a second-order latent variable (Additional file 2) and the SEM analysis (Additional file 3) indicated acceptable fit with the data.

### Measurement model analysis

All variables originally included in the measurement model were retained. As the measurement model indicated a good fit of the data and ‘breakfast’ (0.53 (95% CI 0.47–0.59)), ‘cooked lunch’ (0.68 (95% CI 0.62–0.74)), and ‘cooked food in the evening’ (0.47 (95% CI 0.41–0.53)) loaded significantly on the latent variable ‘meal frequency’ in the measurement model [47]; these variables were found to be appropriate to use as a latent variable. The measurement model also confirmed that the two items for subjective well-being loaded significantly on the latent variable ‘subjective well-being’ (0.74 (95% CI 0.68–0.80) and 0.84 (95% CI 0.77–0.91)) and were therefore chosen to be included in the SEM analysis as a latent variable for well-being.

### Correlation analysis

The correlations presented in Additional file 2 were performed to determine whether the first-order latent variables for the health-related behaviours would load on a second-order latent variable using SEM. The analysis confirmed that the four unhealthy behaviours were significantly correlated with an underlying vulnerability (the second-order latent variable): smoking (0.84), alcohol consumption (0.66), regular meal frequency (−0.51) and engagement in physical activity (−0.36) and were therefore included in the SEM analysis. Meal frequency and physical activity exhibited negative correlations with vulnerability because higher values on these variables reflect regular meal frequency and high physical activity.

### SEM analysis

Path coefficients of direct, indirect and total associations between the variables in the model are presented in full in Additional file 3.

The path coefficients of indirect and total associations in the SEM analysis (Additional file 3) indicated that the individual health-related behaviours were significantly associated with most of the psychosocial conditions. Smoking and alcohol consumption were associated with low levels of well-being and poor social relationships. Smoking was also associated with low self-esteem. Regular meal frequency and physical activity were associated with high levels of well-being and good social relationships. Additional file 3 demonstrates the associations of health-related behaviours with socio-demographic variables (see Indirect and Total associations).

**Fig. 3:**
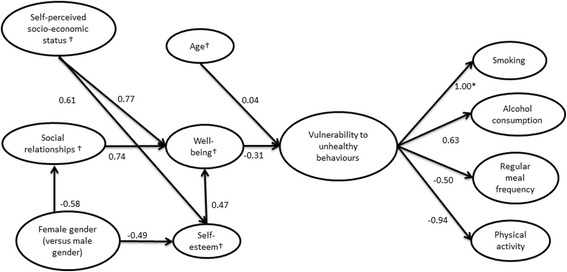
Final path model. This path model, performed with structural equation modelling in LISREL 8.8, presents total path coefficients (both direct and indirect associations) between latent variables (with the hypothesised result outlined in Fig. 1.) First-order latent variables are presented in squares and the second-order latent variable, which was interpreted as a vulnerability to unhealthy behaviours, in the oval circle. Note that the study is cross-sectional and that the direction of causality in the model therefore is unknown. Model fit: *χ* ^2^ 208.31 with df 50, RMSEA 0.08, GFI 0.94, AGFI 0.90 and SRMR 0.08. All path coefficients in the model were statistically significant at 95% confidence intervals except for the path between ‘age’ and ‘vulnerability to unhealthy behaviours’. * The path coefficient between ‘smoking’ and the second-order latent variable was fixed to 1.00 to standardise the second-order latent variable. † Measures high levels versus low levels

The SEM analysis indicated strong significant associations between an underlying vulnerability (the second-order latent variable) and the unhealthy behaviours of smoking (fixed to 1.00), alcohol consumption (0.63), irregular meal frequency (−0.50) and low engagement in physical activity (−0.94) (Additional file 3 and Fig. 3).

A low level of well-being was significantly associated with the underlying vulnerability (−0.31, see Additional file 3 [Direct and Total associations] and Fig. 3). Adolescents whose self-perceived socio-economic status was low (−0.24), female adolescents (0.21), adolescents with poor social relationships (−0.23) and adolescents with lower self-esteem (−0.15) were all significantly associated with vulnerability (see Additional file 3 [Indirect and Total associations]). However, there was no association between higher school grade (age) and vulnerability (see Additional file 3 [Direct and Total associations] and Fig. 3).

Figure 3 and Additional file 3 present the strength of the associations between the psychosocial conditions and socio-demographic variables in the hypothesised model. There were a few strong direct (and total) associations. The association between well-being and good social relationships was strong (0.74). In addition, well-being was strongly related to high self-esteem (0.47). High levels of well-being were associated with high self-perceived socio-economic status (0.77). High levels of well-being were also associated with being male (−0.66) (indirect association). There was a strong association between good social relationships and being male (−0.58), and high self-esteem was related with being male (−0.49) and high socio-economic status (0.61) (direct associations).

## Discussion

A hypothesised model of an underlying vulnerability for a set of unhealthy behaviours in adolescents was investigated using SEM analysis. The SEM model had a good level of fit, indicating that the hypothesised model was supported by the sample data. The SEM analysis demonstrated that well-being and social relationships were associated with all the individual health-related behaviours (smoking, alcohol consumption, meal frequency and physical activity). Smoking was also related with low self-esteem. The findings present that low well-being, poor social relationships, low self-esteem, low self-perceived socio-economic status and being female together represent a vulnerability to the unhealthy behaviours: smoking, alcohol consumption, irregular meal frequency and low level of physical activity. However, older adolescents were not more vulnerable to the unhealthy behaviours than the slightly younger adolescents were, as hypothesised. The hypothesised underlying structure of interrelated psychosocial and socio-demographic variables for unhealthy behaviours was confirmed in the SEM analysis. The study provides a unique insight into the possible framework of psychosocial conditions associated with this set of unhealthy behaviours in adolescents in Sweden. However, this structure needs to be confirmed by longitudinal studies.

The model that was tested in the present study was based mainly on the ‘Individual Health Behaviours Model’ and Morgan’s social capital framework. Similar to the ‘Individual Health Behaviours Model’ [19], our study found low well-being, poor social relationships and low self-esteem to comprise a psychosocial vulnerability towards unhealthy behaviours. Low well-being was also associated with poor social relationships and low self-esteem, also similar to the ‘Individual Health Behaviours Model’. Similar to Morgan’s framework, psychosocial factors analysed in our study were found to be associated with well-being and unhealthy behaviours (Fig. 3, Additional file 3). Earlier studies have identified psychosocial causes for unhealthy behaviours among adolescents too [14, 15, 24, 74], and according to the findings in this study and the findings by Morgan, these factors seem to be important to adolescents’ health-related behaviours in Sweden, England and Spain. A difference in our study design compared with Morgan’s framework is that we investigated adolescents’ psychosocial vulnerability in general and did not investigate it in different contexts (family, neighbourhood, school and peers). Our previous study [75], however, used the same data material as the present study and investigated two of the contexts (school and family) in relation to unhealthy behaviours in adolescents. Morgan argues that these contexts are part of the social capital stocks that comprise the psychosocial vulnerability that underlies low well-being and unhealthy behaviours in adolescents. Another important aspect underlying low well-being and unhealthy behaviours is the interrelation of psychosocial and sociodemographic factors.

Socio-demographic (socio-economic status, gender and age) conditions were included in the hypothesised model that was tested (as based on Morgan’s framework (Fig. 1) [9] and the model by Paulsson-Do et al. [38]). Similar to Morgan’s framework, self-perceived socio-economic status and gender were related to well-being and health-related behaviours. However, in the present study the underlying factors were interrelated unlike the ‘Individual Health Behaviours Model’ and Morgan’s framework, which did not present interrelations between underlying psychosocial and socio-demographic factors of health-related behaviours. It is noteworthy that good psychosocial conditions (good subjective well-being, good psychosocial relationships and high self-esteem) were strongly related to being male in our study (Additional file 3). The study also found that high self-perceived socio-economic status was positively related to well-being and self-esteem indicating that those with good economic conditions have a higher well-being and self-esteem. Longitudinal studies should confirm these findings. The SEM model presented strong associations between well-being, social relationships and self-esteem. Previous studies have also found these associations [10, 12, 13, 52, 53], but have not studied them in relation to vulnerability for unhealthy behaviours.

Our hypothesised model predicted that being female would be associated with poor social relationships. Although this was confirmed by the SEM analysis, earlier studies also suggest that males are more likely to have poor social relationships [49, 76], while Zimmermann [77] found no gender differences. Our finding may be explained by the association with unhealthy behaviours and that unhealthy behaviours seem to be more common in girls than in boys [15, 39, 43]. However, further work on gender differences is needed.

There may be other relationships between the underlying factors than those tested in this study. For example, some research indicates that social relationships between peers can affect adolescents’ self-esteem [76]. These findings, and other possible relationships, may be tested in future studies. The limited number of studies that have examined underlying psychosocial conditions’ potential influence on unhealthy behaviours in general in adolescents are mainly based on American samples. Morgan et al. performed similar studies in Europe along with one recent study, similar to the present study, which presented psychosocial problems associated with several unhealthy behaviours in adolescents [78]. The limited number of studies performed in Europe makes it difficult to compare the present study with previous research performed on European samples, particularly on adolescents in Northern Europe. This is unfortunate because there may be socio-cultural differences between the US and northern European countries, such as Sweden. Rostila [31], for example, found health inequalities between countries with different welfare regimes. Langer and Warheit [79] have argued that factors underlying unhealthy behaviours may differ between socio-cultural settings and Morgan [9] argued for the importance of performing studies in different country contexts. Morgan [9] found that the importance of psychosocial factors to health-related behaviours in adolescents differed in different sociocultural settings. A high quality in degree of social relationships may be very important to the well-being and health-related behaviours of adolescents in countries that value family connectedness (such as the United States or Spain). However, good social relationships could, hypothetically, not be as important to adolescents’ health-related behaviours in a country with a socio-cultural setting that encourages interdependence [37] (such as Sweden). This hypothesis was not confirmed in this study; however, well-being, social relationships and self-esteem were found to be important to the health-related behaviours of Swedish adolescents. Although this has yet to be investigated further, this study concludes that vulnerability in the form of poor psychosocial conditions may be an underlying factor associated with unhealthy behaviours in both Swedish adolescents and Americans (the Individual Health Behaviours Model is based on the general US population, not only adolescents) [19].

This study lays the groundwork for knowledge about how psychosocial conditions are interrelated with each other, with socio-demographic factors and with unhealthy behaviours in northern European adolescents. Further studies should be performed in different cultural contexts to determine and understand further pathways that exist between underlying psychosocial and socio-demographic factors and unhealthy behaviours in adolescents. Longitudinal investigations are needed to draw causal conclusions, however. The next step could be a northern European longitudinal panel design study of 15–16-year-old adolescents to investigate causal interrelationships between psychosocial conditions (well-being, social relationships and self-esteem) and unhealthy behaviours. These studies should include different contexts, such as family, school and peers. Thus, results of these studies’ could identify psychosocial and socio-demographic groups that are in need of extra resources and support.

### Strengths and limitations

This study is one of just a few studies that have analysed the structure of interrelated psychosocial conditions associated with unhealthy behaviours in northern European adolescents. The study did not investigate this in different social contexts, which is a limitation. The study has a representative sample. As this study is cross-sectional, the direction of causality is unknown. Because the data were collected by self-report questionnaires, there is a possibility of response bias. The low variability in answers to some of the questions might have resulted in weaker relationships between some factors than would otherwise have been observed. There may be a disadvantage to use self-report data. A scientific discussion has started regarding how appropriate the measurement of physical activity is when it is performed through self-report among adolescents. This method is very common [80, 81], however, and the question that was used in this study is a well-established and widely used measure for physical activity [80]. Whereas mean replacement was used for the interpersonal distrust and ineffectiveness subscales, there are other options that may also be used for missing values. However, mean replacement is a method often used for the EDI-C subscales [62, 82].

## Conclusions

A psychosocial vulnerability for four common unhealthy behaviours was found in Swedish adolescents. The study presents how well-being, social relationships, self-esteem and socio-demographic factors are interrelated and how these interrelations are associated with unhealthy behaviours in adolescents. The study provides new insights to the field of psychosocial factors to unhealthy behaviours in adolescents in northern Europe. Based on the findings, we suggest the need for a longitudinal panel study of 15–16-year-old adolescents in Northern Europe to investigate causal associations between unhealthy behaviours and low well-being, poor social relationships and low self-esteem. Further studies should be performed in different cultural contexts as well as in different social contexts, such as family and school.

## Additional files

Additional file 1: Table S1. Variables and distribution of answers. (DOCX 25 kb)

Additional file 2: Table S2. Polychoric correlation coefficients of latent variables. (DOCX 16 kb)

Additional file 3: Table S3. Path coefficients of hypothesised direct, indirect and total Structural Equation Modelling associations between latent variables. (DOCX 26 kb)

## Abbreviations

AGFI: Goodness of Fit Index Adjusted for df
GFI: Goodness of Fit Index
EDI-C: Eating Disorder Inventory-Children
RMSEA: root-mean-square error of approximation
SEM: structural equation modelling
SRMR: standardised root-mean-square residual

## References

[1] World Health Organization (1996). Noncommunicable diseases: WHO experts warn against inadequate prevention, particularly in developing countries. Fact sheet no. 106.

[2] World Health Organization (2014). Health for the world’s adolescents - a second chance in the second decade.

[3] Zahra J, Ford T, Jodrell D (2014). Cross-sectional survey of daily junk food consumption, irregular eating, mental and physical health and parenting style of British secondary school children. Child Care Health Dev.

[4] Rampersaud GC, Pereira MA, Girard BL, Adams J, Metzl JD (2005). Breakfast habits, nutritional status, body weight, and academic performance in children and adolescents. J Am Diet Assoc.

[5] Utter J, Scragg R, Mhurchu CN, Schaaf D (2007). At-home breakfast consumption among New Zealand children: associations with body mass index and related nutrition behaviors. J Am Diet Assoc.

[6] Haug E, Rasmussen M, Samdal O, Iannotti R, Kelly C, Borraccino A (2009). Overweight in school-aged children and its relationship with demographic and lifestyle factors: results from the WHO-collaborative health behaviour in school-aged children (HBSC) study. Int J Public Health.

[7] Kelder SH, Perry CL, Klepp KI, Lytle LL (1994). Longitudinal tracking of adolescent smoking, physical activity, and food choice behaviors. Am J Public Health.

[8] Morgan A, Currie C, Due P, Nic Gabhainn S, Rasmussen M, Samdal S, Smith R (2008). Mental well-being in school-aged children in Europe: associations with social cohesion and socioeconomic circumstances. In: social cohesion for mental wellbeing in adolescents. Report of the 2007 WHO/HBSC FORUM.

[9] Morgan A (2011). Social capital as a health assett for young people’s health and wellbeing: definitions, measurement and theory. In division of social medicine.

[10] Gomez-Bustamante EM, Cogollo Z (2010). Predictive factors related to general well-being in adolescent students in Cartagena, Colombia. Rev Salud Publica (Bogota).

[11] Bukowski WM, Hoza B, Boivin M, Laursen B (1993). Popularity, friendship, and emotional adjustment during early adolescence. New directions for child development: close friendships in adolescence.

[12] Smedema SM, Catalano D, Ebener DJ (2010). The relationship of coping, self-worth, and subjective well-being: a structural equation model. Rehabil Couns Bull.

[13] Avci D, Yilmaz FA, Koc A (2012). Correlation between subjective well-being and self-esteem levels of college nursing students. *IAMURE*. International Journal of Social Sciences.

[14] Bergman MM, Scott J (2001). Young adolescents’ wellbeing and health-risk behaviours: gender and socio-economic differences. J Adolesc.

[15] Mazur J, Woynarowska B (2004). Risk behaviors syndrome and subjective health and life satisfaction in youth aged 15 years. Med Wieku Rozwoj.

[16] Wiefferink CH, Peters L, Hoekstra F, Dam GT, Buijs GJ, Paulussen TG (2006). Clustering of health-related behaviors and their determinants: possible consequences for school health interventions. Prev Sci.

[17] Davies LD, Crosby RA, Diclemente RJ, Di Clemente RJ, Santelli JS, Crosby RA (2009). Family influences on adolescent health. Adolescent health – understanding and preventing risk behaviors.

[18] Jessor R, Turbin MS, Frances MC. Predicting developmental change in healthy eating and regular exercise among adolescents in China and the United States: the role of psychosocial and behavioral protection and risk. Journal of Reseach on Adolescence. 2010;20:707–725.

[19] Shi L, Stevens GD (2010). Vulnerable populations in the United States.

[20] Donovan JE, Jessor R, Costa FM (1988). Syndrome of problem behavior in adolescence: a replication. J Consult Clin Psychol.

[21] Donovan JE, Jessor R, Costa FM (1993). Structure of health-enhancing behavior in adolescence: a latent-variable approach. J Health Soc Behav.

[22] Jessor R, Jessor SL (1977). Problem behavior and psychosocial development: a longitudinal study of youth.

[23] Jessor R (1987). Problem-behavior theory, psychosocial development, and adolescent problem drinking. Br J Addict.

[24] Kristjánsson AL, Sigfúsdóttir ID, Allegrante JP (2010). Health behavior and academic achievement among adolescents: the relative contribution of dietary habits, physical activity, body mass index, and self-esteem. Health Educ Behav.

[25] Blum LM, Blum RWM, Di Clemente RJ, Santelli JS, Crosby RA (2009). Resilience in Adolescence. Adolescent health - understanding and preventing risk behaviors.

[26] Lalonde M (1974). A new perspective on the health of Canadians - a working document.

[27] Currie C, Zanotti C, Morgan A, Currie D, de Looze M, Roberts C (2012). Social determinants of health and well-being among young people - health behaviour in school-aged children (HBSC) study: international report from the 2009/2010 survey.

[28] Esping-Andersen G (1990). The three worlds of welfare capitalism.

[29] Taylor-Gooby P, Larsen T, Kananen J (2004). Market means and welfare ends: the UK welfare state experiment. Journal of Social Policy.

[30] Birn AE (2009). Making it politic(al): closing the gap in a generation: healthequity through action on the social determinants of health. Social Med.

[31] Rostila M (2008). Healthy bridges: studies of social capital, welfare, and health.

[32] UNICEF (2007). Child poverty in perspective: an overview of child well-being in rich countries, Innocenti report card 7.

[33] OECD (2009). Doing better for children.

[34] Killoran A, Morgan A, Jagroo J, Killoran A, Kelly MP (2009). Promoting the emotional and social well-being of children in primary education: evidence-based guidance. Evidence-based public health: effectiveness and efficiency.

[35] Rutter MJ, Smith DJ (1995). Psychosocial disorders in young people: time trends and their causes.

[36] Heijmens VJH, van der Ende J, Koot HM, Verhulst FC (2000). Predictors of psychopathology in young adults referred to mental health services in childhood or adolescence. Br J Psychiatry.

[37] Harkness S, Super CM, Rubin K (2006). Themes and variations: parental ethnographies in western cultures. Parenting beliefs, behaviours, and parent-child relations: a cross-cultural perspective.

[38] Paulsson-Do U, Edlund B, Stenhammar C, Westerling R (2014). Vulnerability to unhealthy behaviours across different age groups in Swedish adolescents: a cross-sectional study. Health Psychol Behav Med.

[39] Abudayya AH, Stigum H, Shi Z, Abed Y, Holmboe-Ottesen G (2009). Sociodemographic correlates of food habits among school adolescents (12-15 year) in North Gaza strip. BMC Public Health.

[40] Hoglund D, Samuelson G, Mark A (1998). Food habits in Swedish adolescents in relation to socioeconomic conditions. Eur J Clin Nutr.

[41] Neumark-Sztainer D, Story M, French S, Cassuto N, Jacobs DR, Resnick MD (1996). Patterns of health-compromising behaviors among Minnesota adolescents: sociodemographic variations. Am J Public Health.

[42] Seabra AF, Mendonca DM, Thomis MA, Anjos LA, Maia JA (2008). Biological and socio-cultural determinants of physical activity in adolescents. Cad Saude Publica.

[43] van Nieuwenhuijzen M, Junger M, Velderman MK, Wiefferink KH, Paulussen TW, Hox J, Reijneveld SA (2009). Clustering of health-compromising behavior and delinquency in adolescents and adults in the Dutch population. Prev Med.

[44] Kahn JA, Huang B, Gillman MW, Field AE, Austin SB, Colditz GA (2008). Patterns and determinants of physical activity in U.S. adolescents. J Adolesc Health.

[45] Feingold A (1994). Gender differences in personality: a meta-analysis. Psychol Bull.

[46] Kling KC, Hyde JS, Showers CJ, Buswell BN (1999). Gender differences in self-esteem: a meta-analysis. Psychol Bull.

[47] Cross SE, Madson L (1997). Models of the self: self-construals and gender. Psychol Bull.

[48] Harper JF, Marshall E (1991). Adolescents’ problems and their relationship to self-esteem. Adolescence.

[49] Markiewicz D, Doyle AB, Brendgen M (2001). The quality of adolescents’ friendships: associations with mothers’ interpersonal relationships, attachments to parents and friends, and prosocial behaviors. J Adolesc.

[50] Francis LJ, Jones SH (1996). Social class and self-esteem. J Soc Psychol.

[51] Tsai JL, Ying YW, Lee PA (2001). Cultural predictors of self-esteem: a study of Chinese American female and male young adults. Cultur Divers Ethnic Minor Psychol.

[52] von Rueden U, Gosch A, Rajmil L, Bisegger C, Ravens-Sieberer U (2006). Socioeconomic determinants of health related quality of life in childhood and adolescence: results from a European study. J Epidemiol Community Health.

[53] Miyakawa M, Magnusson Hanson LL, Theorell T, Westerlund H (2012). Subjective social status: its determinants and association with health in the Swedish working population (the SLOSH study). Eur J Pub Health.

[54] Scales PC (1999). Reducing risks and building developmental assets: essential actions for promoting adolescent health. J Sch Health.

[55] Swedish Association of Local Authorities and Regions. Kommungruppsindelning 2011 - Revidering av Sveriges Kommuner och Landstings Kommungruppsindelning. Stockholm: Swedish Association of Local Authorities and Regions; 2010.

[56] The Swedish National Agency for Education. SIRIS - the National Agency for Education´s online information system on results and quality. http://siris.skolverket.se/siris/f?p=SIRIS:164:0::NO:::. Accessed 7 Dec 2017.

[57] The Swedish National Agency for Education. SIRIS - the National Agency for Education´s online information system on results and quality. http://siris.skolverket.se/siris/f?p=SIRIS:147:0::NO:::. Accessed 7 Dec 2017.

[58] Sveriges riksdag. Law 2003:460. https://www.riksdagen.se/sv/Dokument-Lagar/Lagar/Svenskforfattningssamling/Lag-2003460-om-etikprovning_sfs-2003-460/. Accessed 7 Dec 2017.

[59] Diener E, Emmons RA, Larsen RJ, Griffin S (1985). The satisfaction with life scale. J Pers Assess.

[60] Schimmack U, Radhakrishnan P, Oishi S, Dzokoto V, Ahadi S (2002). Culture, personality, and subjective well-being: integrating process models of life satisfaction. J Pers Soc Psychol.

[61] Garner D (1991). The eating disorder inventory-C.

[62] Gustafsson SA, Edlund B, Kjellin L, Norring C (2010). Characteristics measured by the eating disorder inventory for children at risk and protective factors for disordered eating in adolescent girls. Int J Womens Health.

[63] Thurfjell B, Edlund B, Arinell H, Hagglof B, Garner D, Engstrom I (2004). Eating disorder inventory for children (EDI-C): effects of age and gender in a Swedish sample. Eur Eat Disord Rev.

[64] Westerberg-Jacobson J, Edlund B, Ghaderi A (2010). Risk and protective factors for disturbed eating: a 7-year longitudinal study of eating attitudes and psychological factors in adolescent girls and their parents. Eat Weight Disord.

[65] Goodman E, Huang B, Schafer-Kalkhoff T, Adler NE (2007). Perceived socioeconomic status: a new type of identity that influences adolescents’ self-rated health. J Adolesc Health.

[66] Diamantopoulos A, Siguaw J (2007). Introducing LISREL: a guide for the uninitiated.

[67] Jöreskog KG, Sörbom D (1993). LISREL 8: structural equation modeling with the SIMPLIS command language.

[68] Kline RB (2011). Principles and practice of structural equation modeling.

[69] Schumacker RE, Lomax RG (2010). A beginner’s guide to structural equation modelling.

[70] Hensel DJ, Fortenberry JD (2013). A multidimensional model of sexual health and sexual and prevention behavior among adolescent women. J Adolesc Health.

[71] Li X, Stanton B, Yu S (2007). Factorial structure of problem behaviors among urban and rural American adolescents. J Natl Med Assoc.

[72] Turbin MS, Jessor R, Costa FM (2000). Adolescent cigarette smoking: health-related behavior or normative transgression?. Prev Sci.

[73] Hooper D, Coughlan J, Mullen MR (2008). Structural equation modelling: Guidelines for determining model fit. Electronic Journal of Business Research Methods.

[74] James SR., Ellahebokus A, Sewpaul R.,Naidoo P. The association between adolescent risk behaviours and feelings of sadness or hopelessness: a cross-sectional survey of south African secondary school learners. Psychology, Health & Medicine 2017;0:1-12. 10.1080/13548506.2017.1300669 28290218

[75] Paulsson Do U, Stenhammar C, Edlund B, Westerling R (2017). Health communication with parents and teachers and unhealthy behaviours in 15- to 16-year-old Swedes. Health Psychology and Behavioral Medicine.

[76] Berndt TJ, Woodhead M, Faulkner D, Littleton K (1999). Friendship in adolescence. Making sense of social development.

[77] Zimmermann P (2004). Attachment representations and characteristics of friendship relations during adolescence. J Exp Child Psychol.

[78] Busch V, De Leeuw JRJ (2014). Unhealthy behaviors in adolescents: multibehavioral associations with psychosocial problems. Int J Behav Med.

[79] Langer LM, Warheit GJ (1992). The pre-adult health decision-making model: linking decision-making directedness/orientation to adolescent health-related attitudes and behaviors. Adolescence.

[80] Mendonça G, Cheng LA, Nunes-Mélo E, de Farias Júnior JC (2014). Physical activity and social support in adolescents: a systematic review. Health Educ Res.

[81] de Moraes ACF, Guerra PH, Menezes PR (2013). The worldwide prevalence of insufficient physical activity in adolescents; a systematic review. Nutr Hosp.

[82] Westerberg J, Edlund B, Ghaderi A (2008). A 2-year longitudinal study of eating attitudes, BMI, perfectionism, asceticism and family climate in adolescent girls and their parents. Eating Weight Disord.

