# Supplementary material for: Psychosocial vulnerability underlying four common unhealthy behaviours in 15–16-year-old Swedish adolescents: a cross-sectional study
Source: BMC Psychol. 2017 Dec 15;5:39. doi: 10.1186/s40359-017-0209-9 (PMC5732431; doi:10.1186/s40359-017-0209-9)
Supplement: Supplementary file 1 — Variables and distribution of answers. (DOCX 25 kb) [file 40359_2017_209_MOESM1_ESM.docx]

| **Table S1.** Variables and distribution of answers | |  |  |
| --- | --- | --- | --- |
| First-order latent variables: | Indicating variables for first-order latent variables | Answer alternatives: | Frequency (%) |
|  | (number of responses): |  |  |
| Socio-demographic variables:  **Gender** | Gender (492) | Boys | 48.8 |
|  |  | Girls | 51.2 |
| **School grade (age)** | School grade (age) (492) | Grade 8 | 31.1 |
|  |  | Grade 9 | 68.9 |
| **Self-perceived socio-economic status** | How often do you feel that you have | Always; Fairly often | 10.5 |
|  | less money than your peers? (487) | Sometimes | 20.5 |
|  |  | Rather seldom | 27.1 |
|  |  | Never/almost never | 41.9 |
| Psychosocial condition variables:  **Subjective well-being** | How do you feel? (489) | Very bad; Bad | 4.1 |
|  |  | Neither bad or good | 13.1 |
|  |  | Good | 46.2 |
|  |  | Very Good | 36.6 |
|  | How satisfied are you with your life? (490) | Very unsatisfied; Unsatisfied | 4.9 |
|  |  | Neither unsatisfied or satisfied | 15.9 |
|  |  | Satisfied | 44.3 |
|  |  | Very satisfied | 34.9 |
| **Self-esteem** | I feel that I can not do things particularly | Never; Rarely; Sometimes | 80.9 |
|  | well (491) | Often | 11.8 |
|  |  | Usually | 5.5 |
|  |  | Always | 1.8 |
|  | I feel alone (489) | Never; Rarely; Sometimes | 90.6 |
|  |  | Often | 5.7 |
|  |  | Usually | 2.0 |
|  |  | Always | 1.6 |
|  | I feel that I can control things in my life (486) | Always; Usually; Often | 76.5 |
|  |  | Sometimes | 15.6 |
|  |  | Rarely | 5.8 |
|  |  | Never | 2.1 |
|  | I wish I was somebody else (489) | Never; Rarely; Sometimes | 88.1 |
|  |  | Often | 7.4 |
|  |  | Usually | 3.3 |
|  |  | Always | 1.2 |
|  | I feel that I am as good as most | Always; Usually; Often | 67.8 |
|  | adolescents (490) | Sometimes | 20.8 |
|  |  | Rarely | 9.0 |
|  |  | Never | 2.4 |
|  | I like myself (487) | Always; Usually; Often | 65.5 |
|  |  | Sometimes | 21.1 |
|  |  | Rarely | 8.8 |
|  |  | Never | 4.5 |
|  | I do not like myself particularly much (485) | Never; Rarely; Sometimes | 84.9 |
|  |  | Often | 8.0 |
|  |  | Usually | 3.9 |
|  |  | Always | 3.1 |
|  | I feel that I can attain the things I try to | Always; Usually; Often | 75.5 |
|  | do (490) | Sometimes | 20.2 |
|  |  | Rarely | 3.1 |
|  |  | Never | 1.2 |
|  | I feel that I am a good person (488) | Always; Usually; Often | 78.1 |
|  |  | Sometimes | 16.8 |
|  |  | Rarely | 3.5 |
|  |  | Never | 1.6 |
|  | I feel like a bad person (491) | Never; Rarely; Sometimes | 93.1 |
|  |  | Often | 3.3 |
|  |  | Usually | 1.8 |
|  |  | Always | 1.8 |
|  | Total score of self-esteem (492) | Very low | 1.0 |
|  |  | Rather low | 4.3 |
|  |  | Slightly low | 52.4 |
|  |  | High | 42.3 |
| **Social relationships** | I tell others about my feelings (489) | Always; Usually; Often | 34.8 |
|  |  | Sometimes | 33.7 |
|  |  | Rarely | 23.3 |
|  |  | Never | 8.2 |
|  | I trust others (487) | Always; Usually; Often | 71.5 |
|  |  | Sometimes | 18.7 |
|  |  | Rarely | 7.8 |
|  |  | Never | 2.0 |
|  | I talk with other people easily (490) | Always; Usually; Often | 66.7 |
|  |  | Sometimes | 21.8 |
|  |  | Rarely | 10.0 |
|  |  | Never | 1.4 |
|  | I have close friends (490) | Always; Usually; Often | 89.2 |
|  |  | Sometimes | 7.5 |
|  |  | Rarely | 2.6 |
|  |  | Never | 0.7 |
|  | I have difficulties telling others how I | Never; Rarely; Sometimes | 76.1 |
|  | feel (489) | Often | 10.0 |
|  |  | Usually | 8.8 |
|  |  | Always | 5.1 |
|  | I don’t want other people to get to know me | Never; Rarely; Sometimes | 86.0 |
|  | too well (485) | Often | 8.5 |
|  |  | Usually | 3.5 |
|  |  | Always | 2.0 |
|  | I can talk about my personal thoughts and | Always; Usually; Often | 46.8 |
|  | feelings (483) | Sometimes | 31.7 |
|  |  | Rarely | 17.6 |
|  |  | Never | 3.9 |
|  | Total score of psycho-social relations (492) | Very poor | 0.2 |
|  |  | Rather poor | 12.4 |
|  |  | Slightly poor | 66.5 |
|  |  | Good | 20.9 |
| Health-related behavioural variables:  **How often do you eat the** | Breakfast (491) | Rarely/never | 7.9 |
| **following meal during a regular week?** |  | 1–3 days | 13.8 |
| (Meal frequency) |  | 4–6 days | 14.9 |
|  |  | Every day | 63.3 |
|  | Cooked lunch (486) | Rarely/never | 3.7 |
|  |  | 1–3 days | 9.9 |
|  |  | 4–6 days | 28.0 |
|  |  | Every day | 58.4 |
|  | Cooked food in the evening (487) | Rarely/never | 7.0 |
|  |  | 1–3 days | 10.5 |
|  |  | 4–6 days | 17.6 |
|  |  | Every day | 64.9 |
| **Exercise more than 30 minutes** | Exercise more than 30 minutes so that | 0–3 times/month | 13.7 |
|  | you get out of breath or sweat (481) | 1–3 times/week | 44.5 |
|  |  | 4 times/week - every day | 41.8 |
| **Smoking** | Smoking (484) | No (I have never smoked, |  |
|  |  | I have tried, I have stopped) | 75.2 |
|  |  | Yes (I smoke occasionally or daily) | 24.8 |
| **Alcohol consumption** | Alcohol consumption so that you | Never | 52.3 |
|  | become drunk (478) | Less than every second month - |  |
|  |  | about once a month | 35.8 |

Twice a month- more than 4 times/week 11.9
