# Supplementary material for: Psychosocial vulnerability underlying four common unhealthy behaviours in 15–16-year-old Swedish adolescents: a cross-sectional study
Source: BMC Psychol. 2017 Dec 15;5:39. doi: 10.1186/s40359-017-0209-9 (PMC5732431; doi:10.1186/s40359-017-0209-9)
Supplement: Supplementary file 2 — Polychoric correlation coefficients of latent variables. (DOCX 16 kb) [file 40359_2017_209_MOESM2_ESM.docx]

**Table S2.** Polychoric correlation coefficients of second order latent variable and its first order latent variables (indicating variables)

|  | 1. | 2. | 3. | 4. | 5. |  |  |
| --- | --- | --- | --- | --- | --- | --- | --- |
| 1. Meal frequency** | 1.00* |  |  |  |  |  |  |
| 2. Physical activity** | 0.18* | 1.00* |  |  |  |  |  |
| 3. Smoking** | -0.43* | -0.30* | 1.00* |  |  |  |  |
| 4. Alcohol consumption** | -0.34* | -0.24* | 0.97* | 1.00* |  |  |  |
| 5. Vulnerability to unhealthy behaviours*** | -0.51* | -0.36* | 0.84* | 0.66* | 1.00* |  |  |
| Note: |  |  |  |  |  |  |  |
| Polychoric correlation was used |  |  |  |  |  |  |  |
| Standard deviation was set to 1.00 for all coefficients and have a mean of zero (standardized solution) | | | | | | | |
| Fit statistics: *χ*^2^ 163.82 with df 46, RMSEA 0.07, GFI 0.95, AGFI 0.95 and SRMR 0.07 | | | | | | |  |
| * Statistically significant (*p* < 0.05) |  |  |  |  |  |  |  |
| ** First-order latent variable | |  |  |  |  |  |  |
| *** Second-order latent variable interpreted as an underlying vulnerability to unhealthy behaviours | | | | | | |  |
