# Supplementary material for: Psychosocial vulnerability underlying four common unhealthy behaviours in 15–16-year-old Swedish adolescents: a cross-sectional study
Source: BMC Psychol. 2017 Dec 15;5:39. doi: 10.1186/s40359-017-0209-9 (PMC5732431; doi:10.1186/s40359-017-0209-9)
Supplement: Supplementary file 3 — Path coefficients of hypothesised direct, indirect and total Structural Equation Modelling associations between latent variables. (DOCX 26 kb) [file 40359_2017_209_MOESM3_ESM.docx]

| **Table S3.** Path coefficients of hypothesized direct, indirect and total Structural Equation Modelling associations between latent variables | | | | |  |
| --- | --- | --- | --- | --- | --- |
|  |  | Direct association (95% CI)† | Indirect association (95% CI)† | Total association (95% CI)† |  |
| **Second-order latent variable**:** | |  |  |  |  |
|  | Gender (females vs males) |  | 0.21 (0.17 – 0.25)* | 0.21 (0.17 – 0.25)* |  |
|  | Grade (age) (higher vs lower) | 0.04 (-0.01 – 0.09) |  | 0.04 (-0.01 – 0.09) |  |
|  | High socio-economic status**** | | -0.24 (-0.28 – -0.20)* | -0.24 (-0.28 – -0.20)* |  |
|  | High self-esteem |  | -0.15 (-0.22 – -0.08)* | -0.15 (-0.22 – -0.08)* |  |
|  | Good social relationships |  | -0.23 (-0.32 – -0.14)* | -0.23 (-0.32 – -0.14)* |  |
|  | High well-being | -0.31 (-0.36 – -0.26)* |  | -0.31 (-0.36 – -0.26)* |  |
| **Regular meal habits:** |  |  |  |  |  |
|  | Second-order latent variable** | -0.50 (-0.59 – -0.41)* |  | -0.50 (-0.59 – -0.41)* |  |
|  | Gender (females vs males) |  | -0.10 (-0.12 – -0.08)* | -0.10 (-0.12 – -0.08)* |  |
|  | Grade (age) (higher vs lower) |  | -0.02 (-0.03 – -0.01) | -0.02 (-0.03 – -0.01) |  |
|  | High socio-economic status**** | | 0.12 (0.10 – 0.14)* | 0.12 (0.10 – 0.14)* |  |
|  | High self-esteem |  | 0.07 (0.03 – 0.11) | 0.07 (0.03 – 0.11) |  |
|  | Good social relationships |  | 0.12 (0.07 – 0.17)* | 0.12 (0.07 – 0.17)* |  |
|  | High well-being |  | 0.16 (0.14 – 0.18)* | 0.16 (0.14 – 0.18)* |  |
| **Physical activity:** |  |  |  |  |  |
|  | Second-order latent variable** | -0.94 (-1.13 – -0.75)* |  | -0.94 (-1.13 – -0.75)* |  |
|  | Gender (females vs males) |  | -0.20 (-0.24 – -0.16)* | -0.20 (-0.24 – -0.16)* |  |
|  | Grade (age) (higher vs lower) |  | -0.04 (-0.06 – -0.02)* | -0.04 (-0.06 – -0.02) |  |
|  | High socio-economic status**** | | 0.23 (0.19 – 0.27)* | 0.23 (0.19 – 0.27)* |  |
|  | High self-esteem |  | 0.14 (0.07 – 0.21) | 0.14 (0.07 – 0.21) |  |
|  | Good social relationships |  | 0.22 (0.13 – 0.31)* | 0.22 (0.13 – 0.31)* |  |
|  | High well-being |  | 0.30 (0.25 – 0.35)* | 0.30 (0.25 – 0.35)* |  |
| **Smoking:** |  |  |  |  |  |
|  | Second-order latent variable** | 1.00*** |  | 1.00*** |  |
|  | Gender (females vs males) |  | 0.21 (0.17 – 0.25)* | 0.21 (0.17 – 0.25)* |  |
|  | Grade (age) (higher vs lower) |  | 0.04 (0.01 – 0.07) | 0.04 (0.01 – 0.07) |  |
|  | High socio-economic status**** | | -0.24 (-0.28 – -0.20)* | -0.24 (-0.28 – -0.20)* |  |
|  | High self-esteem |  | -0.15 (-0.22 – -0.08)* | -0.15 (-0.22 – -0.08)* |  |
|  | Good social relationships |  | -0.23 (-0.32 – -0.14)* | -0.23 (-0.32 – -0.14)* |  |
|  | High well-being |  | -0.31 (-0.26 – -0.36)* | -0.31 (-0.26 – -0.36)* |  |
| **Alcohol consumption:** |  |  |  |  |  |
|  | Second-order latent variable** | 0.63 (0.52 – 0.74)* |  | 0.63 (0.52 – 0.74)* |  |
|  | Gender (females vs males) |  | 0.13 (0.10 – 0.16)* | 0.13 (0.10 – 0.16)* |  |
|  | Grade (age) (higher vs lower) |  | 0.03 (0.01 – 0.05) | 0.03 (0.01 – 0.05) |  |
|  | High socio-economic status**** | | -0.15 (-0.19 – -0.11)* | -0.15 (-0.19 – -0.11)* |  |
|  | High self-esteem |  | -0.09 (-0.14 – -0.04) | -0.09 (-0.14 – -0.04) |  |
|  | Good social relationships |  | -0.15 (-0.21 – -0.09)* | -0.15 (-0.21 – -0.09)* |  |
|  | High well-being |  | -0.20 (-0.25 – -0.15)* | -0.20 (-0.25 – -0.15)* |  |
| **High self-esteem:** |  |  |  |  |  |
|  | Gender (females vs males) | -0.49 (-0.53 – -0.45)* |  | -0.49 (-0.53 – -0.45)* |  |
|  | Grade (age) (higher vs lower) |  |  |  |  |
|  | High socio-economic status**** | 0.61 (0.57 – 0.65)* |  | 0.61 (0.57 – 0.65)* |  |
|  | Good social relationships |  |  |  |  |
|  | High well-being |  |  |  |  |
| **Good social relationships:** |  |  |  |  |  |
|  | Gender (females vs males) | -0.58 (-0.63 – -0.53)* |  | -0.58 (-0.63 – -0.53)* |  |
|  | Grade (age) (higher vs lower) |  |  |  |  |
|  | High socio-economic status**** | |  |  |  |
|  | High self-esteem |  |  |  |  |
|  | High well-being |  |  |  |  |
| **High well-being:** |  |  |  |  |  |
|  | Gender (females vs males) |  | -0.66 (-0.74 – -0.58)* | -0.66 (-0.74 – -0.58)* |  |
|  | Grade (age) (higher vs lower) |  |  |  |  |
|  | High socio-economic status**** | 0.48 (0.29 – 0.67)* | 0.29 (0.15 – 0.43)* | 0.77 (0.67 – 0.87)* |  |
|  | Good social relationships | 0.74 (0.46 – 1.02)* |  | 0.74 (0.46 – 1.02)* |  |
|  | High self-esteem | 0.47 (0.24 – 0.70)* |  | 0.47 (0.24 – 0.70)* |  |
| Note: |  |  |  |  |  |
| CI: confidence interval |  |  |  |  |  |
| Model fit: *χ*^2^ 208.31 with df 50, RMSEA 0.08, GFI 0.94, AGFI 0.90 and SRMR 0.08 | | |  |  |  |
| * Statistically significant at 95% CI | |  |  |  |  |
| ** The second-order latent variable is hypothesized to represent an underlying vulnerability for unhealthy behaviours. Other variables in this table are | | | | | |
| first-order latent variables. |  |  |  |  |  |
| *** 95% CI could not be measured for this path coefficient because of the standardization of the second-order latent variable which was performed by setting | | | | | |
| the path coefficient to the first-order latent variable ‘smoking’ to 1.00. | | |  |  |  |
| † Empty cells indicates that no measurement was performed for the two variables because an association between them was not included in the | | | | | |
| hypothesized model that was investigated.  **** adolescents´ self-perceived socio-economic status | |  |  |  |  |
